# Development of loop-mediated isothermal amplification for rapid detection of sporotrichosis caused by *Sporothrix schenckii*

**DOI:** 10.14202/vetworld.2023.1356-1362

**Published:** 2023-06-17

**Authors:** Vena Chupia, Jirapat Ninsuwon, Montira Intanon, Surachai Pikulkaew

**Affiliations:** 1 Department of Veterinary Biosciences and Veterinary Public Health, Faculty of Veterinary Medicine, Chiang Mai University, 50100, Thailand; 2 Research Center of Producing and Development of Products and Innovations for Animal Health and Production, Faculty of Veterinary Medicine, Chiang Mai University, Chiang Mai, 50100, Thailand; 3 Veterinary Diagnostic Center, Chiang Mai University Animal Hospital, Faculty of Veterinary Medicine, Chiang Mai University, 50100, Thailand; 4 Department of Food Animal Clinics, Faculty of Veterinary Medicine, Chiang Mai University, 50100, Thailand

**Keywords:** loop-mediated isothermal amplification, molecular detection, rapid detection, *Sporothrix schenckii*, sporotrichosis, visual detection

## Abstract

**Background and Aim:**

*Sporothrix schenckii* is the causative agent of sporotrichosis, which most commonly causes lymphocutaneous infections in immunocompromised hosts. This pathogen infects dogs, cats, cattle, and buffaloes and can potentially infect humans. Diagnosis by fungal culture is lengthy, and although there are several clinical diagnoses and molecular methods, these are complicated and time-consuming for veterinarians. This study aimed to develop a visual diagnostic assay that is less time-consuming and can be used by veterinarians to screen for sporotrichosis.

**Materials and Methods:**

To develop a loop-mediated isothermal amplification (LAMP) assay for sporotrichosis, primers specific for fragments of the 18S rRNA gene of *S. schenckii* were designed. Then, the time and temperature were optimized to successfully achieve LAMP. Ten-fold serial dilutions of DNA were used to determine the detection limit using both LAMP and nested polymerase chain reaction (nPCR) assays.

**Results:**

The optimal LAMP conditions were incubation at 73°C for 30 min. Agarose gel electrophoresis revealed a ladder-like pattern of the LAMP product, and a sky-blue color indicated a positive result. A comparison of the LAMP assay with nPCR revealed that it was 10 times more sensitive than nPCR, with a detection limit of 10 pg. The use of a heat box compared with a thermocycler gave the same results.

**Conclusion:**

Loop-mediated isothermal amplification gives good results and may represent a future alternative diagnostic tool for screening fungal pathogens before the results of conventional fungal cultures are received. However, this method should be further studied to clarify its use with clinical samples.

## Introduction

Sporotrichosis is a disease resulting from fungal infections of the deep skin – subcutaneous mycosis – usually caused by the fungus *Sporothrix schenckii*. This fungus is commonly found in soil, hay, plants, humus, or decaying wood. The infection can be transmitted to pets, such as dogs and cats, as well as cattle and buffaloes, and may also infect humans. *Sporothrix schenckii* enters the body of humans and animals through skin wounds and can also be transmitted through contact with a diseased cat or by scratching [1]. Besides infections in the skin layer, the infection also causes systemic mycosis, with possible infections in the lungs, joints, bones, and brain [2].

*Sporothrix schenckii* is a dematiaceous dimorphic fungus appearing as a dark mold that grows in the mold and yeast phases in different environments [3]. When thriving in soil, plants, decaying wood humus, or in media at 25°C, it grows within 2 weeks. In the first stage, the colony appears as a smooth, white to creamy yeast, which then becomes a darker mold with a sticky and wrinkled surface from the middle of the colony. Microscopically, it appears as a moldy or mycelium form with fibers. Small conidia are arranged in surrounding clusters at the ends of conidiophores, similar to flowers (rosette-like), and single conidia may be found germinating alongside the hyphae [3]. However, when the fungus grows in an animal’s body or is cultured in enriched media incubated at 37°C, the colonies in the yeast phase are smooth and creamy to brown in color. The microscopic characteristics include budding yeast cells with an oval shape. Sporotrichosis is diagnosed by fungal culturing of samples taken for clinical examination on Sabouraud dextrose agar (SDA) [3]. The fungus grows as a discrete colony, but it takes at least 7 days to seed the fungus for identification. Since immunological methods have not yet been associated with a specific species, molecular identification methods have been developed for diagnosis. These DNA-based methods can identify at the species level, which reduces diagnostic time and improves the specificity, sensitivity, and accuracy of different methods. Polymerase chain reaction (PCR)-based methods, such as restriction fragment length polymorphism (RFLP), random amplified polymorphic DNA (RAPD), internal transcribed spacer (ITS) DNA sequencing, PCR targeting the topoisomerase II gene, amplified fragment length polymorphisms (AFLP), and M13 PCR fingerprinting, can be used for fungal identification. Alternatively, loop-mediated isothermal amplification (LAMP), which, unlike the above-mentioned PCR-based methods, does not require expensive equipment or tools, is an easy technique for infection diagnosis [4]. It can be applied in small laboratories, and the turnaround time is <1 day.

This study aimed to develop a method of diagnosis for *S. schenckii* that is faster than fungal culturing, the gold standard method, which takes a long time to perform. We achieved this using LAMP. Our method is quick, very precise, and can be performed using inexpensive scientific tools. If our LAMP method is further developed, it will benefit the veterinary treatment of animals infected with *S. schenckii* and could limit disease progression. Furthermore, it will help with intervention to prevent pet owners and other animals from contracting the disease.

## Materials and Methods

### Ethical approval

All procedures related to fungus manipulation in this study were approved by the Institutional Biosafety Committee, Chiang Mai University, Chiang Mai, Thailand (CMUIBCA-0763010).

### Study period and location

The study was conducted from July 31, 2020, to July 30, 2022, at the Laboratory Diagnostic Center of Faculty of Veterinary Medicine, Chiang Mai University, Thailand.

### Fungal culture and species confirmation using PCR

The fungus obtained from the Laboratory of Veterinary Diagnostic and Laboratory Center, Faculty of Veterinary Medicine, Chiang Mai University, Thailand was cultured on SDA (BD Difco™, Franklin Lakes, NJ, US) and incubated at 25°C for 2 weeks. For the dimorphic fungal test, we subcultured the fungus from the above culture onto brain heart infusion agar and incubated at 37°C for approximately 1 week. The yeast phase was colonized, smooth, and creamy to brown in color. Microscopic observation showed budding yeast cells with an oval shape.

Polymerase chain reaction and sequencing were performed to confirm that the fungus was *S. schenckii*. The DNA of fungus from the 2-week SDA culture was extracted using a PureDireX^®^ genomic DNA isolation kit (Bio-Helix, Taiwan). The DNA concentration and purity were determined spectrophotometrically (Du730, Beckman Coulter^®^, Brea, CA, USA). and the samples were stored at −20°C. Polymerase chain reaction was performed using primer pair ITS1 (5’-TCC GTA GGT GAA CCT GCG G-3’) and ITS4 (5’-TCC TCC GCT TAT TGA TAT GC-3’), which amplify a region in the 18S RNA gene [5]. Each PCR reaction was performed in a final volume of 25 μL containing 5 μM of each primer, 2× Quick Taq DyeMix (TOYOBO, Osaka, Japan), and 2 μL of DNA template (100 ng of DNA). The thermal cycling conditions were as follows: 94°C (2 min); 40 cycles f 94°C (30 s), 58°C (30 s), and 72°C (30 s); and final extension at 72°C (7 min). The product size was approximately 599 base pairs, which was determined by 1.5% agarose gel electrophoresis in Tris-borate-ethylene diamine tetraacetic acid (TBE) buffer (Biotechnology Inc., Gyeonggi-do, Korea). The images were captured using a GelMax™ 148 imager (ultra-violet products, Cambridge, UK). The PCR product was sequenced and compared with the results of DNA sequences (GenBank accession number: MH729029.1) in the National Center for Biotechnology and Information (NCBI) database using Basic Local Alignment Search Tool (BLAST) (https://blast.ncbi.nlm.nih.gov/blast.cgi).

### Nested PCR (nPCR)

The extracted DNA of *S. schenckii* was analyzed by nPCR targeting the 18S rRNA gene region [6]. Polymerase chain reaction reactions were performed in a final reaction volume of 20 μL. The outer primer pair was SS1 (5’-CTC GTT CGG CAC CTT ACA CG-3’) and SS2 (5’-CGC TGC CAA AGC AAC GCG GG-3’). The inner primer pair was SS3 (5’-ACT CAC CAG GTC CAG ACA CGA TG-3’) and SS4 (5’-CGC GGG CTA TTT AGC AGG TTA AG-3’). The primer pairs were synthesized on an oligonucleotide synthesizer (Bio Basic Inc., Canada). The first round of PCR was performed using 2 μL of extracted DNA (100 ng), 0.6 μL each of primers SS1 and SS2 (0.24 mM), and 10 μL KOD One™ PCR Master Mix (TOYOBO). The thermal cycling conditions included an initial PCR activation step at 94°C (2 min); 40 cycles at 94°C (30 s), 58°C (30 s), and 72°C (30 s); and a final extension step at 72°C for 7 min. The reaction components of the second PCR round were the same as the first PCR round, except that 2 μL of the first round PCR product and 0.6 μL of the inner primer pair SS3 and SS4 (0.24 μM) were used. The thermal cycling conditions were 30 cycles at 98°C (10 s) and 68°C (2 s). The PCR product was determined by 1.5% gel electrophoresis in TBE buffer (Biotechnology Inc., Gyeonggi-do, Korea)). The images were captured using a GelMax™ 148 imager (ultra-violet products).

### Loop-mediated isothermal amplification

#### Loop-mediated isothermal amplification primer design

**Figure-1:**
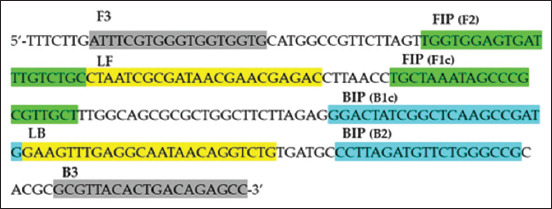
Primer recognition site of Loop-mediated isothermal amplification (LAMP) primer in Target DNA site of primer annealed with target DNA. The four necessary LAMP primers (F3, FIP [F2 and F1c], BIP [B1c and B2], and B3) create a dumbbell structure, and the two loop primers, LF and LB, accelerate the LAMP reaction.

The LAMP Primer was designed using the published sequence of *S. schenckii* (GenBank accession no. M85053) and PrimerExplorer V5 software (Eiken Chemical Co., Ltd.) (http: primerexplorer.jp/lampv5e/index.html). Six LAMP primers were designed according to previously described criteria by Notomi *et al*. [4] and Tomita *et al*. [7]: One forward inner primer, one backward inner primer, two outer primers (F3 and B3), and two loop primers (LF and LB). The primer binding sites and sequences are shown in Table-1 and Figure-1.

**Table-1:** LAMP primers used in this study.

LAMP primers	Sequence (5→3’)
FIP	AGCAACGCGGGCTATTTAGCATTTTTTTGGTGGAGTGATTTGTCTGC
BIP	GGACTATCGGCTCAAGCCGATGTTTTTTCGGCCCAGAACATCTAAGG
F3	ATTTCGTGGGTGGTGGTG
B3	GGCTCTGTCAGTGTAACGC
LF	GTCTCGTTCGTTATCGCGATTAG
LB	GAAGTTTGAGGCAATAACAGGTCTG

LAMP=Loop-mediated isothermal amplification

#### Optimization of LAMP reaction

Different temperature and time conditions were tested to optimize the LAMP reaction. The reaction was performed in a final volume of 25 μL (LavaLAMPTM DNA Master Mix, Lucigen^®^, USA). The temperatures tested were 63°C, 65.2°C, 67.8°C, 70.5°C, 72.9°C, and 74°C, and time varied from 10 to 60 min. The samples were heated to 80°C for 2 min to stop the reaction. The LAMP reaction products were visualized using 1.5% agarose gel electrophoresis. The gel was stained with RedSafe™ (iNtRon Biotechnology Inc., Korea), nucleic acid staining solution, revealing a ladder-like pattern (i.e., multiple bands of different molecular weights).

#### Detection and confirmation of LAMP products

The detection limit and specificity were investigated after determining the optimal time and temperature for our LAMP assay. To study the detection limit, serial 10-fold dilutions of *S. schenckii* genomic DNA were tested from 8–10 g to 10–13 g using 1.5% agarose gel electrophoresis and compared colorimetrically using hydroxynaphthol blue (HNB). The detection limit was determined using thermocycler and heat box. To determine the accuracy, three different pathogens that infect the skin of animals were used: *Microsporum canis*, *Microsporum gypseum*, and *Trichophyton mentagrophytes*.

## Results

### Fungal culture and species confirmation using PCR

**Figure-2:**
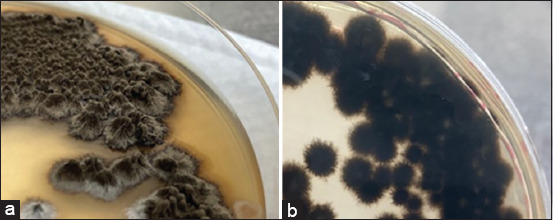
Colonies of *Sporothrix schenckii*: (a) Front side and (b) reverse side. In the dimorphic fungal test, the fungus changed from the mold stage to a yeast colony, with a smooth, oily skin, and a cream or brown color (Figure-3). Under microscopic examination, the yeast cells were found to have a round to oval shape.

**Figure-3:**
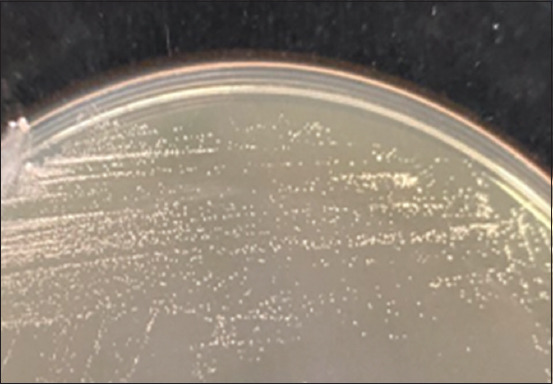
The yeast form of *Sporothrix schenckii* on brain heart infusion agar.

From the macroscopic examination, we saw that the first stage of the colony was smooth, resembling white to creamy yeast. Later, it darkened to black and became sticky and wrinkled from the middle of the colony. On the reverse side of the colony, the edge was faded and the middle was dark in color (Figure-2) and the yeast stage was confirmed (Figure-3). Regarding the microscopic examination characteristics, it appeared in a mycelium form with branching fibers. Spores were formed on conidiophores perpendicular to moldy fibers. Small spores or conidia were arranged in groups surrounding the ends of conidiophores. Rosette-like and single conidia were found growing from the sides of the hyphae.

The fungal species was confirmed molecularly through PCR using primers specific to a partial sequence in the small subunit of the ribosomal RNA gene (18S rRNA gene). The PCR product comprised approximately 599 base pairs. The sequencing data were compared with sequences from the NCBI database using BLAST, revealing that the sequences were *S. schenckii*.

### Nested PCR

**Figure-4:**
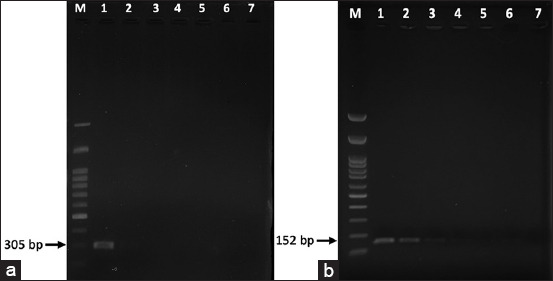
The sensitivity of the nested polymerase chain reaction (PCR) assay with DNA of *Sporothrix* *schenckii*. (a) First-round and (b) second round PCR assay. Lane M: DNA marker (100-bp ladder); lane 1 = 10 ng, lane 2 = 1 ng, lane 3 = 0.1 ng, lane 4 = 10 pg, lane 5 = 1 pg, lane 6 = 0.1 pg and lane 7: Negative control.

Nested PCR using the primers of Hu *et al*. [6] revealed that the detection limit was 10 ng of target DNA in the first round of PCR and 0.1 ng in the second round (Figure-4).

### Loop-mediated isothermal amplification

**Figure-5:**
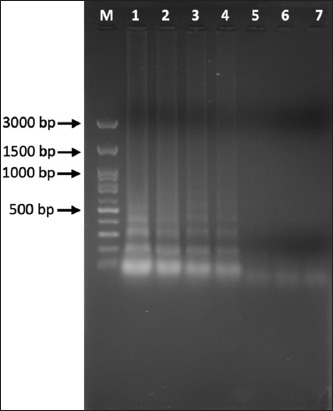
Detection limit of loop-mediated isothermal amplification (LAMP) using the set of LAMP primers. Lane M: DNA marker(100-bp ladder); lane 1 = 10 ng, lane 2 = 1 ng, lane 3 = 0.1 ng, lane 4 = 10 pg, lane 5 = 1 pg, lane 6 = 0.1 pg and lane 7: Negative control.

**Figure-6:**
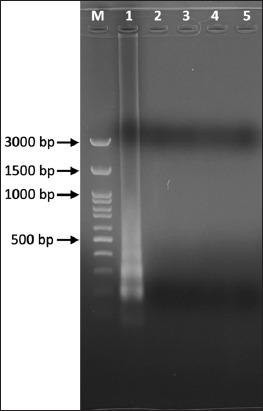
Specificity test of the loop-mediated isothermal amplification. Lane M: DNA marker; lane 1: *Sporothrix* *schenckii*; lane 2: *Microsporum canis*; lane 3: *Microsporum gypseum*; lane 4: T. mentagrophytes; lane 5: Negative control.

The optimal temperature and time for the LAMP technique applied to *S. schenckii* was 72.9°C for 30 min. Regarding the detection limit, the LAMP method clearly detected 0.01 ng of template DNA (Figure-5), while the detection limit of nPCR was 0.1 ng of template DNA (Figure-4). In the specificity test, both the LAMP and PCR methods correctly identified *S. schenckii*, and no amplification products were detected in the assays using the three other pathogens (*M. canis*, *M. gypseum*, and *T. mentagrophytes*) or in the negative control. These findings were inferred from gel electrophoresis (Figure-6).

**Figure-7:**
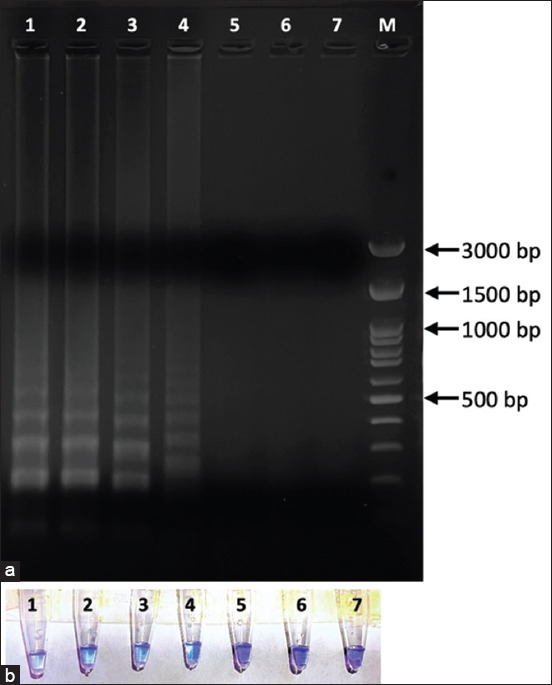
Detection limit of Loop-mediated isothermal amplification using heat box the result was shown by (a) gel electrophoresis and (b) hydroxynaphthol blue indicator. Lane 1: 10 ng; lane 2: 1 ng; lane 3: 0.1 ng; lane 4: 10 pg; lane 5: 1 pg; lane 6: 0.1 pg; lane 7: negative control and lane M: DNA marker.

In addition, HNB was used to detect LAMP products and compared with the agarose gel electrophoresis results. Furthermore, the use of a thermocycler was compared with a heat box. Hydroxynaphthol blue was used at a concentration of 120 ng, and its limit of detection of the DNA template was 0.01 ng (Figure-7) for both the thermocycler and the heat box, implying that the results derived from gel electrophoresis were the same irrespective of whether the thermocycler or heat box was used.

## Discussion

The fungi present in our environment cause diseases in humans and animals, especially those that are immunocompromised, weak, or have a skin injury. In addition, the risk of fungal exposure is higher in farmers, who are vulnerable to thorns or wounds, and in animals with a mischievous nature that like to dig into the ground and play in the water. In companion animals, dermatophytes in healthy rabbits and cats [8, 9] and sporotrichosis in cats [10] can lead to zoonosis and cause disease in humans, especially those who are immunocompromised. *Sporothrix schenckii* is commonly found in rotting soil, plants, or vegetables in tropical, hot, and humid locations, such as Thailand. The *S. schenckii* complex consists of *S. schencki*i *sensu strictu*, *Sporothrix brasiliensis*, *Sporothrix globosa*, *Sporothrix mexicana*, and *Sporothrix luriei*. The infection causes diseases in humans and animals and usually results in chronic mycotic infection, especially in the cutaneous and subcutaneous tissues, and in the lymphoid system. Humans and animals are often infected through wounds and skin abrasions [11, 12].

The standard method for diagnosing sporotrichosis is a fungal culture [13], but this is time-consuming and requires at least 2 weeks. The primary screening method is a microscopic examination, which directly detects pathogens from lesion samples but has a low sensitivity [2]. In addition, faster diagnostic methods, such as RFLP, RAPD, ITS DNA sequencing, PCR targeting the topoisomerase II gene, AFLP, and PCR fingerprinting, have been developed for sporotrichosis diagnosis [14–18]. However, these molecular techniques require specific and expensive equipment, and the turnaround time is at least 3–4 h, making them difficult to apply in small laboratories or some small animal hospitals.

Loop-mediated isothermal amplification is a good method for diagnosing veterinary diseases because of its speed and accuracy [19]. Therefore, LAMP assays have been developed and applied in early screening tests, such as for *Leishmania* protozoa [20, 21], *Nosema ceranae* in honeybees [22, 23], and *Ichthyophthirius multifiliis* protozoa in cyprinid fish [24], as well as the detection of *Penicillium marneffei* in tissues [25], *Trichosporon asahii* detection in clinical samples [26], and bovine tuberculosis in dairy cattle [27].

We developed a LAMP method to detect *S. schenckii* that resulted in effective detection at very low concentrations (10 pg) through both gel electrophoresis and when using HNB as an indicator. Furthermore, the results were the same irrespective of whether a thermocycler or a heat box was used. Using nPCR based on the same set of primers [6], the detection limit of the first round was 10 ng, and the second round was 0.1 ng. In contrast, the detection limit of our LAMP assay was 0.01 ng, which was less than the amount that could be detected using nPCR. However, Hu *et al*. [6] reported that the detection limit of nPCR using this same set of primers was 4 fg, which is approximately 10,000-fold lower than our LAMP assay. The sensitivity results of nPCR in our study differ from those of Hu *et al*. [6], which may be because different PCR kits were used, and the strain of *Sporothrix* spp. and the PCR conditions differed in each study.

In studies using PCR, such as Liu *et al*. [28], the PCR detection limit for *S. schenckii* was 5 pg, which differs from the results of Rodrigues *et al*. [29], who reported a PCR detection limit of 10 fg. In addition, some studies comparing the results of LAMP with nPCR showed that the LAMP methods had a lower detection limit than nPCR [30, 31] and PCR [32], while others reported the same detection limit (result) for LAMP and nPCR [33]. This may be because the primer set used for LAMP and the LAMP kit was different, which may have affected the quantity and concentration of substances, thereby affecting the reaction time and temperature.

Since the LAMP method can be used to detect infection in a preliminary sample, it can be applied in the field. It does not require expensive equipment and has high accuracy and sensitivity. Some studies use HNB in LAMP reactions [34], allowing the detection of the LAMP product with the naked eye immediately after the LAMP reaction. As such, Le and Vu [35] showed that LAMP can be used to detect many microorganisms, such as bacteria, viruses, and fungi, and found that contamination massively affects the final result. Contamination may occur during the preparation of the master mix, due to an overly long incubation time (causing false positive results), or during the loading of LAMP products for gel electrophoresis, which can cause product contamination between wells.

## Conclusion

Our LAMP assay for *S. schenckii* represents an alternative method for screening for pathogens before the results of the fungal culture are received. The LAMP technique is easy to perform, and there is no need for special equipment (a heat box can be used instead of a thermocycler). Our assay also has a low detection limit and a short turnaround time. Therefore, our colorimetric LAMP method is suitable not only for fungal laboratory research but also for clinical diagnosis of infectious diseases in the field. However, the sample detection should be developed to reduce the time required, aiding veterinarians in rapid diagnosis and establishing the necessary approach to treatment, prevention, and control before the disease is transmitted to humans.

## Authors’ Contributions

VC and SP: Project administration, conceptualization, and original draft preparation. VC, MI, and JN: Methodology, investigation, and validation. VC: Data curation, formal analysis, and visualization. SP: Project administration, conceptualization, and original draft preparation. All authors have read, reviewed, and approved the final manuscript.
